# Modeling difference x-ray scattering observations from an integral membrane protein within a detergent micelle

**DOI:** 10.1063/4.0000157

**Published:** 2022-10-31

**Authors:** Daniel Sarabi, Lucija Ostojić, Robert Bosman, Adams Vallejos, Johanna-Barbara Linse, Michael Wulff, Matteo Levantino, Richard Neutze

**Affiliations:** 1 Department of Chemistry and Molecular Biology, University of Gothenburg, Box 462, 40530 Gothenburg, Sweden; 2 European Synchrotron Radiation Facility, 38043 Grenoble Cedex 9, France

## Abstract

Time-resolved x-ray solution scattering (TR-XSS) is a sub-field of structural biology, which observes secondary structural changes in proteins as they evolve along their functional pathways. While the number of distinct conformational states and their rise and decay can be extracted directly from TR-XSS experimental data recorded from light-sensitive systems, structural modeling is more challenging. This step often builds from complementary structural information, including secondary structural changes extracted from crystallographic studies or molecular dynamics simulations. When working with integral membrane proteins, another challenge arises because x-ray scattering from the protein and the surrounding detergent micelle interfere and these effects should be considered during structural modeling. Here, we utilize molecular dynamics simulations to explicitly incorporate the x-ray scattering cross term between a membrane protein and its surrounding detergent micelle when modeling TR-XSS data from photoactivated samples of detergent solubilized bacteriorhodopsin. This analysis provides theoretical foundations in support of our earlier approach to structural modeling that did not explicitly incorporate this cross term and improves agreement between experimental data and theoretical predictions at lower x-ray scattering angles.

## INTRODUCTION

An accurate measurement of protein conformational changes as they evolve with time has long been a goal of structural biology.^1^ Serial crystallography approaches, first implemented at x-ray free electron lasers^2^ (XFELs), have revolutionized the field of time-resolved crystallography since numerous technical challenges that limited the sphere of application of time-resolved Laue diffraction were overcome.^3–5^ The critical benefit of time-resolved crystallography is that a successful study delivers high-resolution structural insight into the chemical details of an evolving reaction from sub-picoseconds to minutes. Recent developments in single-particle cryo-electron microscopy can also provide structural insight into the progress of a biological reaction at high-resolution,^6,7^ and real-time NMR studies of protein dynamics have yielded insights into protein unfolding and refolding.^8^ As methods for characterizing protein conformational changes become increasingly mainstream, a full appreciation of the relationship between structure and function must increasingly incorporate structural dynamics.

Although time-resolved crystallography is undergoing a period of rapid growth,^4^ a fundamental limitation of time-resolved crystallographic techniques is that samples must both crystallize and remain in a highly ordered arrangement as the reaction evolves. It is not always possible to recover well diffracting crystals for any chosen system of study and a reaction of interest may be slowed in the crystalline state.^4,9,10^ Moreover, crystallographic methods cannot visualize protein conformational changes that are incompatible with the crystal packing lattice since they are either suppressed or they disrupt the crystalline order. Time-resolved x-ray solution scattering (TR-XSS) studies of protein structural changes have been developed to address these limitations.^11–29^ The key advantage of TR-XSS is that it provides a relatively direct measurement of the timescale and number of species with distinct secondary structural characteristics. Conversely, since the sample is a randomly oriented ensemble of protein molecules in solution, the measured information is only one-dimensional. As such, TR-XSS is not as information rich as time-resolved x-ray diffraction and structural modeling relies upon low-resolution shape reconstructions^22,26,27^ or leans heavily upon additional information that may be extracted from crystallographic structures^11,12,15,18,25^ or molecular dynamics trajectories.^13–15,21,23,24,27–29^

Structural interpretation of TR-XSS data recorded from integral membrane proteins^12,18,25,28,29^ is further complicated by the fact that a detergent micelle, which surrounds the integral membrane protein,^30^ frequently contains more atoms than the protein itself and is highly disordered. Consequently, difference x-ray scattering data extracted from TR-XSS experiments rely upon structural fluctuations within the detergent micelle averaging out due to the very large number of molecules, which are exposed to an x-ray beam in any experiment. For example, at a concentration of 0.5 mM, approximately 10^12^ protein molecules are found in a 3.6 nl sample volume, which is the volume calculated from an x-ray beam 60 × 120 *μ*m^2^ passing through a 500 *μ*m^2^ capillary.^12^ It is, however, not computationally feasible to perform simulations of 10^12^ membrane-protein/micelle systems, and therefore, approximations are required. In our earlier modelling,^12,18,24,25^ we argued that the influence of the membrane protein-micelle cross term on the x-ray scattering differences do not compromise the structural conclusions drawn from x-ray scattering difference data in the domain 0.2 Å^−1^ ≤ q ≤ 1.0 Å^−1^. Here, we examine these assumptions more critically by fitting TR-XSS data from bacteriorhodopsin (bR)^12^ against structures extracted by combining structural results from time-resolved x-ray crystallography^31,32^ and molecular dynamics simulations that explicitly include a detergent micelle. This analysis highlights the benefits and shortcomings of our earlier approach and provides theoretical foundations for extending the structural analysis of TR-XSS data to a larger set of integral membrane proteins.

## THEORY

For any sample consisting of j atoms, the x-ray scattering from the sample is given by summing the x-ray scattering contributions from each of the atoms of the sample with a phase-factor associated with the spatial location of each atom relative to an arbitrary origin,^33^

$$F\left(q\right)=\sum_{j}f_{j}\;\exp(iq\cdot r_{j}),$$ (1)

where q is a 3D vector in reciprocal space corresponding to the change in momentum of the scattered x-ray and has a magnitude q=4π sin θ/λ; λ is the x-ray wavelength; θ is half the angle through which the x-ray beam is deflected; $F\left(q\right)$ is a complex number called a structure factor that is specified for all values of q; $f_{j}$ is the atomic scattering factor of atom j of the sample; and $r_{j}$ is the 3D coordinate in real space of atom j. The intensity of the scattered x-ray beam for any vector q is proportional to the magnitude squared of the structure factor,

$$S\left(q\right)={\left|F\left(q\right)\right|}^{2}=\sum_{n=1}^{N}{\left|F_{n}\left(q\right)\right|}^{2}.$$ (2)

If samples are assumed to be monodisperse in solution, it is possible to average over all random orientations of molecules to recover,

$$S\left(q\right)={N\langle |F\left(q\right)|}^{2}\rangle ,$$ (3)

where ⟨⋯⟩ denotes rotational averaging in space and yields an isotropic x-ray scattering distribution (i.e., depends only upon the magnitude of q). When summing over all N molecule in the sample, we have made the assumption that the interference between separate molecules may be neglected.^33^ Several packages have been written which build upon this formula to analyze x-ray scattering data. In this work, we utilize the package CRYSOL^34^ for x-ray scattering calculations. CRYSOL evaluates Eq. (3) using the atomic coordinates of macromolecules as input and a utilizes a multipole expansion to calculate the spherically averaged scattering pattern.

**FIG. 1.**
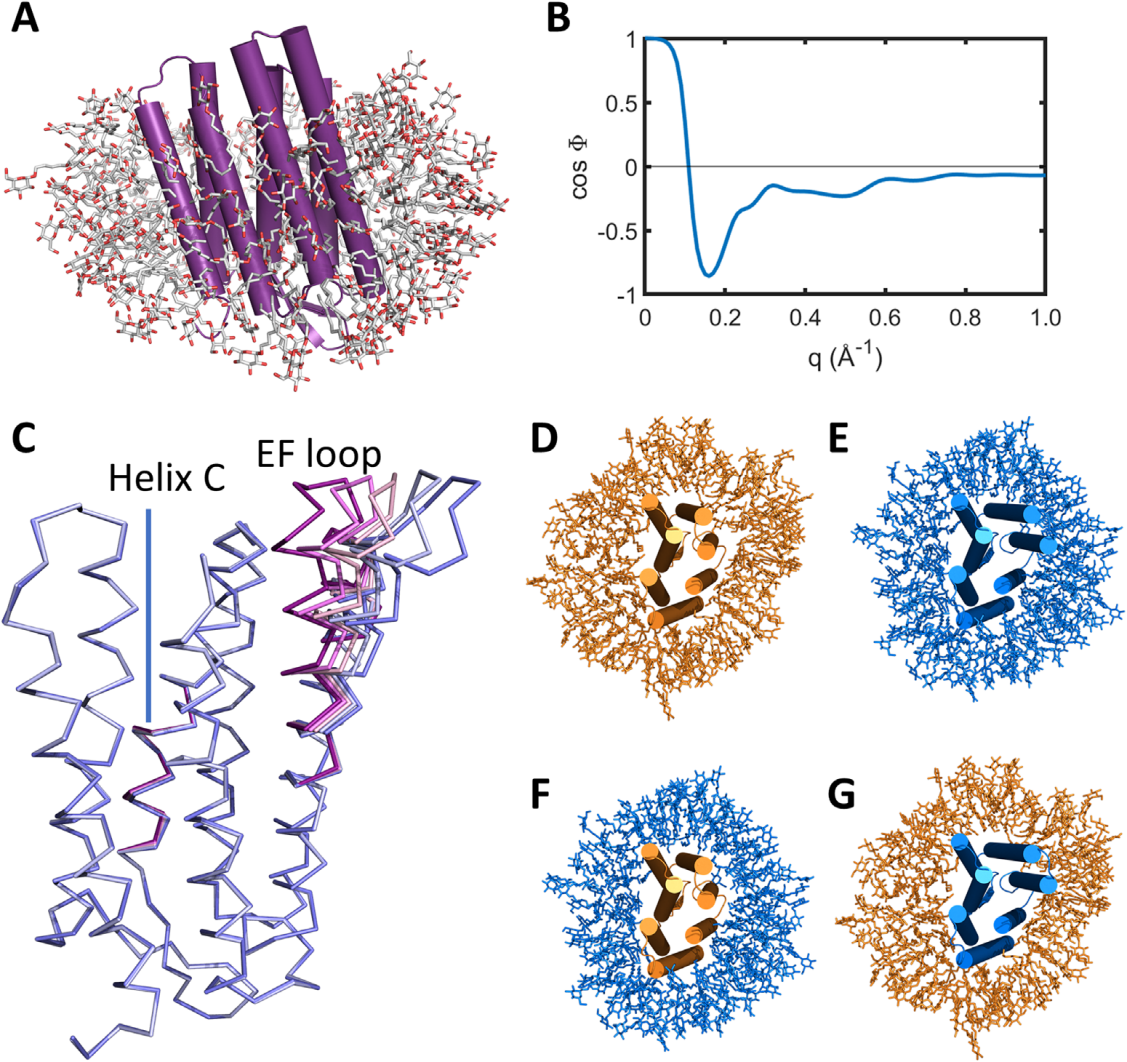
Molecular dynamics simulations of bacteriorhodopsin within a detergent micelle. (a) Structure of bR within a detergent micelle consisting of 190 molecules of β-octyl glucoside. (b) Plot of the term $\text{cos }\Phi_{pm}(q)$ as defined in Eq. (6), which indicates the relative phase coherence between the x-ray scattering from the protein and detergent micelle: $\text{cos }\Phi_{pm}\left(q\right)=1$ means the x-ray scattering is in phase; $\text{cos }\Phi_{pm}(q)=-1$ is out of phase; and $\text{cos }\Phi_{pm}(q)\;\to 0$ indicates little phase coherence. (c) Movements of Cα-atoms of helix C from Pro77 to Pro 91 extracted from pdb entries 5B6Z minus 5B6V; and movements of Cα-atoms for helices E and F from Leu152 to Pro186 extracted from pdb entries 6RPH minus 6RQP, with adjustments in the loop region. These coordinates were then used as targets for molecular dynamics simulations using GROMACS according to Eq. (12). (d) A snapshot from a GROMACS simulation of the resting conformation of bR within a detergent micelle. (e) A snapshot from a GROMACS simulation of the light-activated conformation of bR within a detergent micelle. (f) Creation of a pdb file with a snapshot of the protein from the resting conformation trajectory placed within a snapshot of the micelle from the light-activated conformation trajectory. (g) Creation of a pdb file with a snapshot of the protein from the light-activated trajectory placed within a snapshot of the micelle from the resting conformation trajectory.

When working with integral membrane proteins surrounded by a detergent micelle, we can divide the protein and micelle contributions of Eq. (1) to become

$$F_{pm}\left(q\right)=F_{p}\left(q\right)+F_{m}\left(q\right),$$ (4)

for which $F_{p}\left(q\right)$ represents the structure factor calculated from the protein alone; $F_{m}\left(q\right)$ represents the structure factor calculated from the detergent micelle alone; and $F_{pm}\left(q\right)$ is the structure factor calculated from both combined. Substituting Eq. (4) into Eq. (3) and performing rotational averaging over samples in solution, and noting that ${\left\langle |F\left(q\right)\right|}^{2}\rangle$ is a real number, gives

$$\begin{array}{c} \left\langle {\left|F_{pm}\left(q\right)\right|}^{2}\rangle ={\left\langle |F_{p}\left(q\right)+F_{m}\left(q\right)\right|}^{2}\right\rangle \\ ={\left\langle |F_{p}\left(q\right)\right|}^{2}\rangle +\langle {\left|F_{m}\left(q\right)\right|}^{2}\rangle \\ +2\text{cos}\Phi_{pm}\left(q\right)\sqrt{\left\langle {\left|F_{p}\left(q\right)\right|}^{2}\right\rangle }\sqrt{\left\langle {\left|F_{m}\left(q\right)\right|}^{2}\right\rangle } \end{array},$$ (5)

where this expansion is used to define a new variable, $\Phi_{pm}(q)$, which we introduce to in order to separate the protein and detergent micelle scattering terms within this formalism. As such, $\text{cos}\Phi_{pm}(q)$ can be evaluated by inverting Eq. (5) and utilizing Eq. (3) to recover,

$$\text{cos}\Phi_{pm}\left(q\right)=\frac{S_{pm}\left(q\right)-S_{p}\left(q\right)-S_{m}\left(q\right)}{2\sqrt{S_{p}\left(q\right)}\sqrt{S_{m}\left(q\right)}},$$ (6)

where $S_{p}\left(q\right)$ is the isotropic solution scattering intensity calculated from the protein alone, $S_{m}\left(q\right)$ is the isotropic solution scattering intensity calculated from the detergent micelle alone, and $S_{pm}\left(q\right)$ is the isotropic solution scattering intensity calculated from both protein and micelle combined. In practice, we calculate $\text{cos }\Phi_{pm}\left(q\right)$ by writing separate pdb files containing all of the atoms of the system (Fig. 1), another containing the atoms of the protein alone, and another containing the atoms of the detergent micelle alone. We then calculate the x-ray solution scattering intensities from the corresponding pdb files using CRYSOL. A representative example of $\text{cos }\Phi_{pm}\left(q\right)$ calculated from bacteriorhodopsin in the detergent β-octyl glucoside [Fig. 1(a)] is given in Fig. 1(b). It can be seen that $\text{cos }\Phi_{pm}\left(q\right)$ varies smoothly with q between the values 1 and −1; $\text{cos }\Phi_{pm}\left(0\right)$ is unity due to the fact that all scattering in the forward direction is in phase; and at higher scattering angles, $\text{cos }\Phi_{pm}\left(q\right)\to 0$ as the x-ray scattering from the atoms of the protein and micelle lose phase coherence.

In TR-XSS studies, the most important experimental observation is the change in x-ray scattering as a function of x-ray scattering angle and time, ${\Delta S}_{\mathrm{expt}}(q,\Delta t)$. When modeling protein conformational changes, we are, therefore, concerned with how changes in the structure of the integral membrane protein lead to changes in the measured x-ray scattering amplitudes. From Eq. (5), and using the notation $S_{p}\left(q\right)$ in favor $N{\left\langle |F_{p}\left(q\right)\right|}^{2}\rangle$, etc…, we find that if structure of the membrane protein or detergent micelle changes then corresponding change in x-ray scattering intensities will be

$$\begin{array}{c} {\Delta S}_{pm}\left(q\right)={\Delta S}_{p}\left(q\right)+{\Delta S}_{m}\left(q\right)+2\;\text{cos}\;\Phi_{pm}\left(q\right)(\sqrt{S_{p}\left(q\right)}\Delta \sqrt{S_{m}\left(q\right)}\\ +\sqrt{S_{m}\left(q\right)}\Delta \sqrt{S_{p}\left(q\right)})+2\sqrt{S_{p}\left(q\right)}\sqrt{S_{m}\left(q\right)}\;\Delta \left(\text{cos}\;\Phi_{pm}\left(q\right)\right)\\ \approx {\Delta S}_{p}\left(q\right)\left(1+\frac{\sqrt{S_{m}(q)}}{\sqrt{S_{p}}(q)}\text{cos }\Phi_{pm}(q)\right)\\ +{\Delta S}_{m}\left(q\right)\left(1+\frac{\sqrt{S_{p}(q)}}{\sqrt{S_{m}}(q)}\text{cos }\Phi_{pm}(q)\right)\\ +2\sqrt{S_{p}\left(q\right)}\sqrt{S_{m}\left(q\right)}\;\Delta \left(\text{cos }\;\Phi_{pm}\left(q\right)\right), \end{array}$$ (7)

where ${\Delta S}_{p}\left(q\right)$ is the difference between the x-ray scattering predictions generated from one protein structural model (the candidate structure) and the initial structural model (the resting conformation) and ${\Delta S}_{m}\left(q\right)$ is the difference between the x-ray scattering predictions generated from one micelle structural model and another. Likewise, $\Delta \left[\text{cos}\;\Phi_{pm}\left(q\right)\right.]$ is calculated as the change in $\text{cos}\;\Phi_{pm}(q)$ predicted by Eq. (6) when comparing two different molecular conformations of the entire system. We also used the chain rule to make the approximation $\Delta \sqrt{S_{p}(q)}\approx \Delta S_{p}(q)/[2\sqrt{S_{p}\left(q\right)}]$ and $\Delta \sqrt{S_{m}(q)}\approx \Delta S_{m}(q)/[2\sqrt{S_{m}\left(q\right)}$], which hold if $\Delta \sqrt{S_{p}(q)}\ll S_{p}(q)$ and ${\Delta S}_{m}(q)\ll S_{m}(q)$, both of which are reasonable assumptions for any TR-XSS study of protein structural changes.

The three terms of Eq. (7) illustrate the challenge of analyzing TR-XSS studies of integral membrane proteins. The first term, ${\Delta S}_{p}\left(q\right)[1+\frac{\sqrt{S_{m}(q)}}{\sqrt{S_{p}(q)}}\text{cos }\Phi_{pm}(q)]$, is proportional to x-ray scattering changes arising from conformational changes in the protein but is modulated due to the scattering cross term between the protein and membrane. Likewise the second term, ${\Delta S}_{m}\left(q\right)[1+\frac{\sqrt{S_{p}(q)}}{\sqrt{S_{m}(q)}}\text{cos }\Phi_{pm}(q)]$, is proportional to the x-ray scattering changes associated with structural changes within the detergent micelle and is also modulated due to the scattering cross term between the protein and membrane. The final term, $2\sqrt{S_{p}\left(q\right)}\sqrt{S_{m}\left(q\right)}\;\Delta [\text{cos }\;\Phi_{pm}\left(q\right)]$, reflects how changes in the positions of the atoms of the protein and the micelle influence the effective phase coherence of x-ray scattering cross term between the protein and micelle. In our previous analyses^12,18,24,25^ we effectively assumed that both ${\Delta S}_{m}(q)$ and $\Delta [\text{cos }\Phi_{pm}\left(q\right)]$ were vanishingly small outside of the small-angle scattering domain and, therefore, only the first term of Eq. (7) needed to be considered during structural fitting. In this work we use the analysis of multiple conformations sampled in molecular dynamics trajectories of the protein within a detergent micelle to assess the validity of these assumptions and, consequently, to extend the modeling formalism as appropriate.

When comparing theoretical predictions with experimental data, it is also essential to determine the influence of the solvent volume displaced by both the protein and micelle, which is usually referred to as the solvent-excluded volume.^33^ If $F_{\mathrm{solv}}\left(q\right)$ denotes the x-ray scattering from the solvent-excluded volume due to the presence of the protein and detergent micelle then we can write the total x-ray scattering after correcting for the solvent-excluded volume, $F_{\mathrm{tot}}\left(q\right)$, as

$${\left|F_{\mathrm{tot}}\left(q\right)\right|}^{2}={\left|F_{pm}\left(q\right)-F_{\mathrm{solv}}\left(q\right)\right|}^{2},$$ (8)

where the negative sign preceding $F_{\mathrm{solv}}\left(q\right)$ arises from the fact that the x-ray scattering from the solvent-excluded volume must be subtracted. Limiting ourselves to consider only isotropic solution x-ray solution scattering from randomly oriented molecules [Eq. (3)] and applying the same logic as above, allows us to recover,

$$S_{\mathrm{tot}}\left(q\right)=S_{pm}\left(q\right)+S_{\mathrm{solv}}\left(q\right)-2\text{cos}\;\Phi_{\mathrm{solv}}\sqrt{S_{pm}\left(q\right)}\sqrt{S_{\mathrm{solv}}\left(q\right)},$$ (9)

which, in turn, defines

$$\text{cos }\Phi_{\mathrm{solv}}\left(q\right)=\frac{S_{pm}\left(q\right)+S_{\mathrm{solv}}\left(q\right)-S_{\mathrm{tot}}\left(q\right)}{2\sqrt{S_{pm}\left(q\right)}\sqrt{S_{\mathrm{solv}}\left(q\right)}}.$$ (10)

When predicting x-ray scattering changes as an integral membrane protein changes conformation, we cannot reliably predict the changes in structure of the solvent-excluded volume. We instead assume that both the resting and altered conformations are equally modulated by solvent-exclusion effects and Eq. (9) can then be used to calculate the ratio $S_{\mathrm{tot}}(q)/S_{pm}(q)$ for all values of q. With this rationale, we make the approximation that the theoretical predictions, ${\Delta S}_{pm}\left(q\right)$, calculated from Eq. (7) should be scaled according to

$${{\Delta S}_{\mathrm{theory}}\left(q\right)=\Delta S}_{pm}\left(q\right)\times \frac{S_{\mathrm{tot}}(q)}{S_{pm}(q)},$$ (11)

in order to compare theoretical predictions against experimental difference x-ray scattering data.

## RESULTS

### TR-XSS data collection

**FIG. 2.**
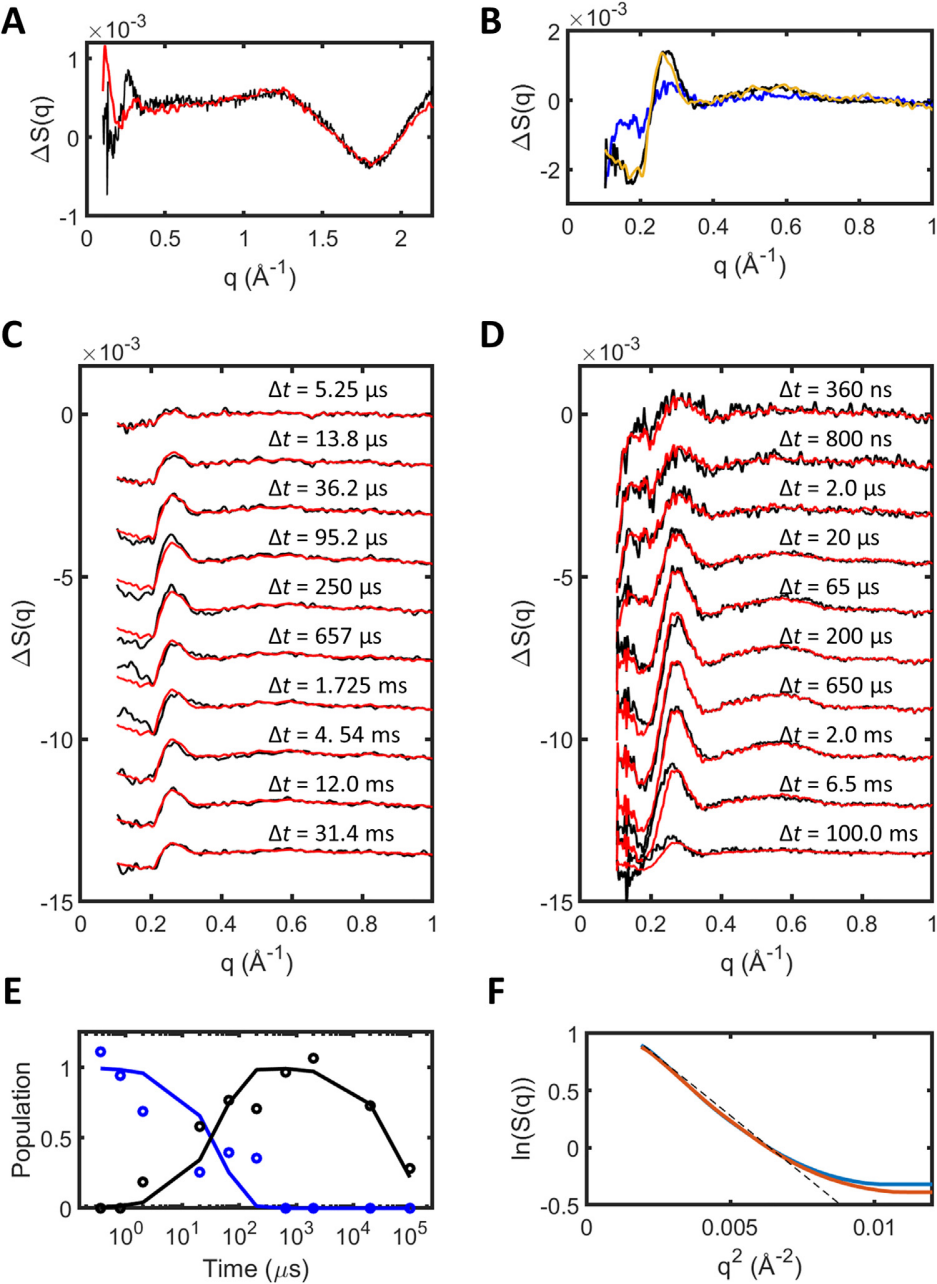
TR-XSS data recorded from detergent solubilized samples of bacteriorhodopsin. (a) Infrared light induced changes in x-ray scattering (red line, characteristic of sample heating) superimposed upon the difference XSS curve recorded for Δ*t *=* *100 ms (black line, previously taken to indicate the influence of heating^12^). (b) Difference XSS basis spectra extracted from TR-XSS data after heat removal. Basis spectra extracted for state 2 from data from 2009 (black line) and 2017 (mustard line) are in strong agreement, whereas the state 1 basis spectra (blue line) were extracted from only the 2009 data. (c) TR-XSS data recorded in 2017 after heat removal. (d) TR-XSS data from 2009 after heat removal. Both datasets used the IR illuminated curves from 2017 (a) for heat-removal. (e) Time-scale for the growth and decay of states 1 (blue line) and 2 (black line) from the 2009 data after heat removal. Circles show the populations for the two states recovered in a linear fit to each time-point. (f) Guinier plot, $\ln[S\left(q\right)]\;\mathrm{vs}.\;q^{2}$, of the total x-ray scattering data at two protein concentrations (35 mM, blue; 25 mM, red). The departure from linearity at low- q suggests some degree of protein aggregation, although this interpretation may be complicated by the presence of a lipid environment.^30,50^

Detergent solubilized samples of bR were prepared as previously described.^12^ TR-XSS data (Fig. 2) were recorded at the beamline ID09 of the ESRF using polychromatic x-rays following photo-excitation with a green (532 nm) laser pulse 4 ns in duration. TR-XSS data were recorded for the time delays Δ*t *=* *5.25, 13.8, 36.2, 95.2, 250, 657 *μ*s, 1.725, 4.54 12.0, and 31.4 ms between the arrival of the 532 nm photoactivation laser pulse and the x-ray probe. Results from these measurements were compared with earlier TR-XSS measurements from bR^12^ also recorded using a 532 nm laser pulse for photoactivation but 100 ns in duration, and for the time-delays Δ*t *=* *360, 800 ns, 2, 20, 65, 200, 650 *μ*s, 2, 20, and 100 ms. In our previous TR-XSS studies, we used a physical translation of the goniometer to translate samples between each laser flash,^12^ whereas in these studies, we flowed the sample through a capillary and thereby replaced the sample between each and every laser flash. In this work, we further recorded XSS data during the photo-excitation of bR samples using continuous illumination with an infrared (IR) laser with a wavelength of 1470 nm, which allowed samples of solubilized bR to be heated without initiating the bR photocycle. In this manner, we recorded directly the influence of sample heating on different XSS spectra [Fig. 2(a)].

In our earlier analysis,^12^ we assumed that the time-delay Δ*t *=* *100 ms represented the difference spectrum, ΔS(*q*), arising purely from the effects of heating. This assumption, however, was an approximation since a superposition of the difference scattering data from 2009 for the 100 ms time-point, ΔS_2009_(*q*, Δ*t* = 100 ms), with the difference scattering recorded here using IR excitation, ΔS_2017_(*q*, IR), shows a significant discrepancy for *q *<* *0.3 Å^−1^ [Fig. 2(a)]. We, therefore, removed laser-induced heating from the difference XSS data by subtracting ΔS_2017_(*q*, IR) from both the 2017 and 2009 data, where the scaling factor for α is chosen to minimize the difference x-ray scattering $\sum \left({\left|\Delta S(q,\;\Delta t)-\alpha \cdot {\Delta S}_{2017}(q,\;\mathrm{IR})\right|}^{2}\right)$ over the domain $0.51\;{Å}^{-1}\leq q\leq 1.45\;{Å}^{-1}$. The resulting heat-corrected time-dependent difference XSS data, ${\Delta S}_{HC}\left(q,\;\Delta t\right),$ are shown in Figs. 2(c) and 2(d), respectively. Linear decomposition of the 2009 data after the removal of heating produced two basis spectra [Fig. 2(b)] with the populations of components 1 and 2 crossing at Δt  ≈  35 *μ*s [Fig. 2(e)]. Given the limited time-sampling of these data, this value is close to the timescale for the crossing of two populations (Δt  ≈  13 *μ*s) in a time-resolved serial femtosecond crystallography (TR-SFX) study using microcrystals of bR^31^ and which correlated well with the L-to-M spectral transition. Unexpectedly, the amplitude of the difference XSS signal after the removal of heat from the 2017 data was only 40% of the amplitude achieved in the 2009 data. As such, linear decomposition of the TR-XSS difference data from 2017 failed to produce a convincing difference signal for the first component, but the difference XSS signal for the second component was in close agreement (Pearson correlation coefficient of 97%) with that recovered for component 2 from the 2009 data [Fig. 2(b)]. This reproducibility demonstrates that different sample preparations, which show variations in detergent composition as well as other experimental parameters, do not unduly influence the experimental difference XSS signal.

The choice of an appropriate pump laser fluence in time-resolved diffraction and x-ray scattering studies has received considerable attention.^4,35,36^ In these studies, the pump laser fluence and protein concentration differed between the 2009 and 2017 data collection runs. The earlier studies passed 250 *μ*J/pulse through a spot focused to a FHWM (full width half maximum) of 265 × 265 *μ*m^2^, amounting to an average pump laser fluence across the FWHM of 227 mJ/cm^2^. Using the procedure described in Ref. 24, we estimate a laser-induced temperature jump ΔT = 0.5 °C from the observed difference XSS signal. Since the protein concentration was 0.57 mM, this heat jump equates to approximately 15 photons being absorbed per bR molecule,^4^ which is 14% of the dimensionless product σ ⋅ F/hν = 105,^4^ where σ is the resting-state cross section of bR, F is the average fluence through the FWHM, h is Planck's constant, and ν is the frequency of the absorbed photon. In the 2017 data-collection, 500 *μ*J/pulse were focused into a laser spot FWHM of 310 *μ*m × 1700 *μ*m, which equates to an average pump laser fluence of 60 mJ/cm^2^ yielding σ ⋅ F/hν = 28. Given that the protein concentration was 1.3 mM, the measured laser induced heating of 0.25 °C equates to three photons being absorbed per bR molecule, or 11% of σ ⋅ F/hν. That these heating estimates are 14% and 11% of the value anticipate from σ ⋅ F/hν, respectively, may be due to the optical density of the sample through a 1 mm capillary (OD ≈ 1.3 over 0.5 mm, 10^−1.3^ = 0.05, 2009 data) and a 500 *μ*m capillary (OD ≈ 1.5 over 0.25 mm, 10^−1.5^ = 0.02, 2017 data) causing the laser fluence throughout the probed sample volume to be much lower than at the surface of the capillary. It is, however, difficult to explain why the amplitude of the difference signal extracted from the 2017 study [Fig. 2(b)] is only 34% of the difference signal observed in 2009,^12^ given that the protein concentration in the latter experiment was more than twice that of the earlier study, and therefore, the occupancy of the excited state population recorded in 2017 was only 15% of that achieved in the 2009 data. It is possible that the difference photo-kinetics of the photo-stationary phase (data were collected in 2009 using a 100 ns pump-laser;^12^ data were collected in 2017 using a 5 ns pump-laser) may underpin this observation. A Guinier plot calculated for the absolute x-ray scattering at two protein concentrations [Fig. 2(f)] also shows a departure from linearity, which may indicate sample aggregation. It may, therefore, be counterproductive to push the protein concentration to the point where the optical density of the sample across the flow-cell becomes too large. We, thus, recommend that, in addition to optimizing the spatial overlap between the laser focus and the x-ray beam, time should also be taken to optimize other parameters such as the protein concentration, the flow-cell thickness, the pump laser fluence, and (whenever possible) the pump-laser duration before proceeding to collect TR-XSS data.

### Influence of the detergent micelle on difference x-ray scattering

When modeling difference x-ray scattering basis spectra [Fig. 2(b)] extracted from heat-corrected TR-XSS data [Figs. 2(c) and 2(d)], it is necessary to predict x-ray scattering changes for a set of candidate secondary structural conformational changes. Disagreement between these predictions and the experimental difference spectra is then minimized by sampling various amplitudes and combinations of the chosen motions. From the theoretical developments above, Eq. (7) highlights in addition to the influence of protein conformational changes on the x-ray scattering, $\Delta S_{p}(q)$, it is important to also model the influence of changes within the micelle on the difference x-ray scattering, $\Delta S_{m}(q)$, and changes in the phase relationships between the x-ray scattering from the protein and from the detergent micelle, $\Delta \left[\text{cos }\Phi_{pm}\left(q\right)\right.]$. We approach this problem by performing molecular dynamics simulations of bacteriorhodopsin placed within a detergent micelle of β-octyl glucoside [Fig. 1(a)] using the package GROMACS.^37,38^ Candidate motions of α-helices were extracted from published time-resolved serial crystallography studies of bacteriorhodopsin,^31,32^ and energy restraints of 1000 kJ/nm^2^ were used to drive the protein conformational states of bacteriorhodopsin toward the Cα backbone coordinates describing these candidate motions. Specifically, a vector describing Cα movements within helix C ${(\Delta C\alpha }_{C})$ was extracted from pdb entry 5B6Z minus 5B6V using Cα coordinates from Pro77 to Pro91; a vector describing Cα movements within helices E and F ${(\Delta C\alpha }_{EF})$ was extracted from pdb entry 6RPH minus 6RQP using Cα coordinates from Leu152 to Pro186; and a perturbation of Cα atoms from Leu211 to Phe219 of helix G associated with retinal isomerization was also incorporated ${(\Delta C\alpha }_{G})$. Moreover, electron density for the EF loop for entry 6RPH was inspected, and for those residues where the electron density was not well defined (residues 157, 158, and 161–167), the ${\Delta C\alpha }_{EF}$ displacements were determined from a weighted average of the coordinate displacements of the adjacent residues. The amplitudes of helices E and F and the helix C motions were then scaled by the variables γ and δ, respectively, and the Cα atoms of bacteriorhodopsin were driven toward the coordinates,

$${C\alpha }_{bR}\to \;{C\alpha }_{bR}+{{\gamma \times \Delta C\alpha }_{EF}+\delta \times \Delta C\alpha }_{C}+{\Delta C\alpha }_{G},$$ (12)

using energy restraints of 1000 kJ/nm^2^ during molecular dynamics simulations [Fig. 1(c)] of 20 ns (preparative analysis) or 5 ns (fitting runs) in duration using a time step of 2 fs, with pdb files written out for x-ray scattering calculations every 10 ps. Each of these simulations contained 3565 atoms within the protein of which 1816 atoms are hydrogen atoms and 1749 are non-hydrogen atoms; and the micelle contained 190 molecules of β-octyl glucoside with 9120 atoms of which 5320 are hydrogen atoms and 3800 are non-hydrogen atoms. Because the retinal is isomerized for all activated state trajectories, we did not scale ${\Delta C\alpha }_{G}$ by a variable parameter but rather we kept the amplitude of this motion equal to the change observed using time-resolved x-ray crystallography. Although this assumption could be challenged, the inclusion of too many free parameters during refinement against the 1D difference x-ray scattering data also risks overfitting.

Since the x-ray scattering contribution is proportional to ${\langle \left|F_{p}\left(q\right)\right|}^{2}\rangle$, the scattering contribution of the detergent micelle is approximately five times larger than that of the protein. Moreover, of the 231 residues within the bR model, only 59 residues (15 in helix C + 35 in helices EF + 9 in helix G) had their backbone Cα atoms displaced (i.e., 26% of the protein) according to Eq. (12). As such, we are aiming to predict the impact on the x-ray scattering of Cα movements associated with approximately 26% of the protein, or 8% of the protein plus micelle system. Moreover, whereas molecular dynamics simulations restrain the Cα atoms of the protein, there were no such restraints associated with the atoms of the detergent micelle. As such, to utilize the predictions of Eq. (7), it was essential to develop a strategy for which x-ray scattering changes associated with random fluctuations of the detergent micelle did not completely dominate the predicted changes.

**FIG. 3.**
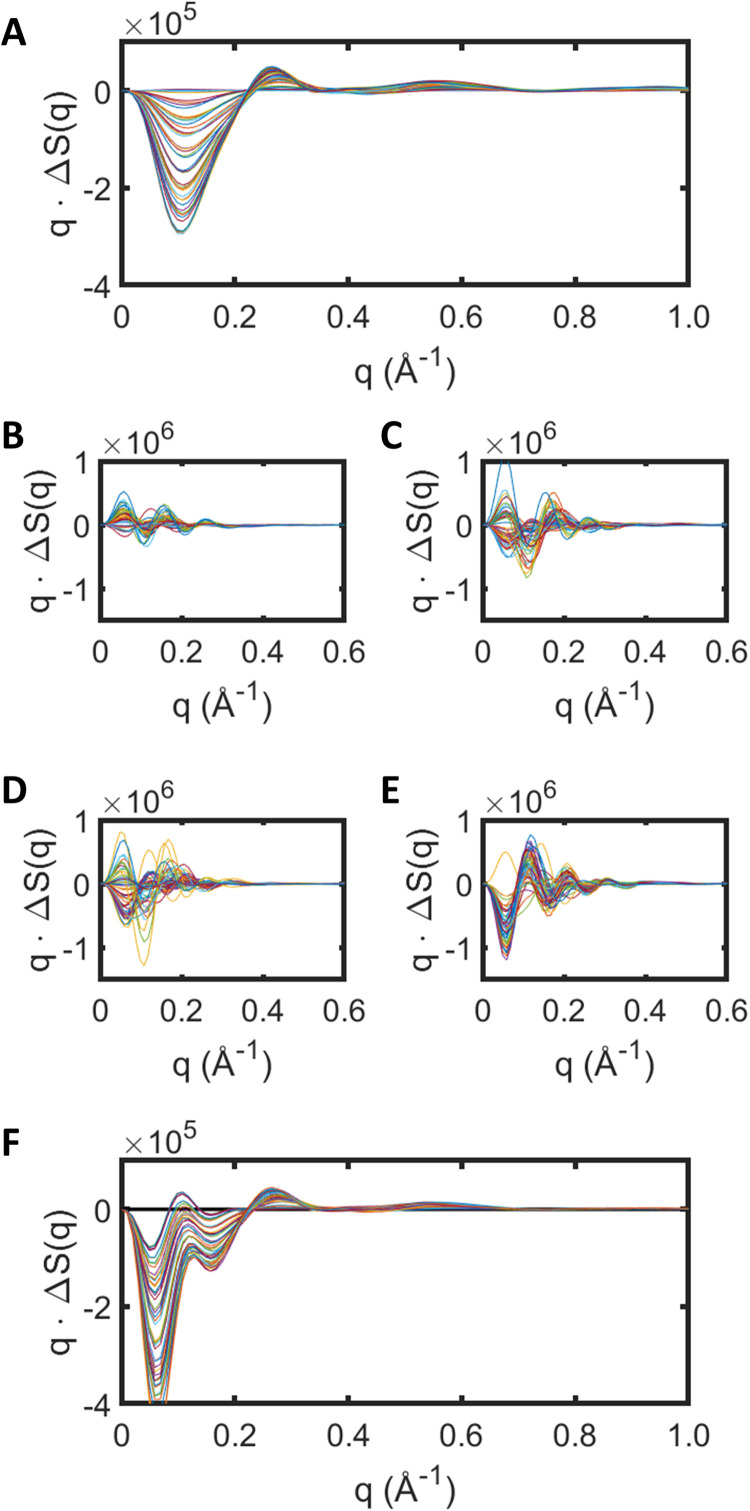
Difference x-ray scattering predictions from molecular dynamics trajectories of bR placed within a detergent micelle formed by 190 molecule of β-octyl glucoside. (a) Difference x-ray scattering predictions from the protein alone as γ and δ vary according to Eq. (12). X-ray scattering changes, $\Delta S_{p}(q)$, evolve smoothly with as γ and δ. These curves are calculated from 2000 snapshots from a trajectory 20 ns in duration. (b)–(e) Difference x-ray scattering predictions extracted from the same molecular dynamics trajectories from the micelle alone. (b) is calculated from the first 500 snapshots of the trajectory; (c) is calculated from the next 500 snapshots; (d) is calculated from the next 500 snapshots; and (e) is calculated from the last 500 snapshots of the 20 ns trajectory. These results show little correlation of $\Delta S_{m}\left(q\right)$ with the values of γ and δ and change significantly as the simulation evolves, reflecting the random nature of structural changes within the micelle. (f) Difference x-ray scattering predictions from the protein plus micelle system, $\Delta S_{pm}(q)$, calculated using Eq. (13). As with (a), the interchange the detergent micelle coordinated extracted from parallel simulations [Figs. 1(f) and 1(g)] yields x-ray scattering changes that evolve smoothly as γ and δ are varied.

X-ray scattering changes (without solvent correction) arising from CRYSOL calculations from the protein in isolation, $\Delta S_{p}(q)$ of Eq. (7), are shown in Fig. 3(a) as γ is varied stepwise from 0 to 1.5 in ten steps of $\frac{1}{6}$ and δ is varied from 0 to $\frac{4}{3}$ in five steps of $\frac{1}{3}$. Despite fluctuations associated with individual conformations along these Cα-restrained molecular dynamics trajectories, when the principal SVD components of the difference x-ray scattering intensities are extracted from the set of 2001 difference x-ray scattering curves (calculated pairwise, by sampling a perturbed and resting conformation every 100 fs of their respective trajectories), there is a smoothly varying change in the predicted difference x-ray scattering as γ and δ are varied. This demonstrates that the structural bias imposed by Eq. (12) during Cα-restrained molecular dynamics simulations is sufficiently powerful to allow quantitative estimates of γ and δ values when fitting against the experimental difference x-ray scattering spectra. As such the use of energy restraints to drive Cα atoms toward a given target structure extends our earlier modeling of these difference x-ray scattering data using rigid motions of α-helices.^12^ Similarly, conformational ensembles extracted from molecular dynamics simulations have been used to fit TR-XSS data from visual rhodopsin^25^ and several other approaches using molecular dynamics have also been reported.^13–15,21,23,24,27–29^

In contrast with the x-ray scattering predictions deriving from the protein in isolation, difference x-ray scattering predictions extracted from the same set of molecular dynamics trajectories but focusing upon changes in x-ray scattering resulting from the micelle alone [the term proportional to $\Delta S_{m}(q)$ in Eq. (7)] do not predict smoothly evolving changes that correlate with γ and δ. Whereas there are similarities in the period of oscillations in the predicted difference x-ray scattering curves which correlate inversely with the physical dimensions of the detergent micelle, the amplitude and sign of the predicted difference x-ray scattering curves fluctuates throughout the course of the simulations [Figs. 3(b)–3(e)]. This issue arises because there are no restraints on the motion of detergent molecules within the micelle and therefore every trajectory may depart significantly from the starting micelle structure. Moreover, when longer simulations of 100 ns were explored, there was no evidence that the system settled to a steady-state equilibrium. It is thus too computationally expensive to perform sufficiently long simulations in order to average out these fluctuations. Nevertheless, although the difference x-ray scattering curves describing the influence of the term $\Delta S_{m}(q)$ in Eq. (7) do not predict smoothly evolving changes that correlate with γ and δ, it is possible to use SVD analysis of the fluctuating $\Delta S_{m}(q)$ curves and thereby extract the principal SVD component, which may be incorporated as a low-angle correction to the predicted difference x-ray scattering.

In addition to structural changes within the protein and within the detergent micelle, we must also consider changes in the x-ray scattering cross term between the protein and the detergent micelle as the protein changes structure, summarized in Eq. (7) as the term involving $\Delta [\text{cos }\Phi_{pm}\left(q\right)]$ This cross term is influenced by structural changes in both the protein and the micelle as γ and δ are varied [Fig. 1(c)]. To side-step the issue of structural fluctuations within the detergent micelle [Figs. 3(b)–3(e)], we developed the following protocol to extract the influence of $\Delta [\text{cos }\Phi_{pm}\left(q\right)]$ on $\Delta S_{pm}(q)$ arising from conformational changes within the protein alone. As illustrated in Fig. 1, we first generate coordinate files extracted from molecular dynamics trajectories of the protein in its resting conformation *(* $p_{\mathrm{rest}})$, within a detergent micelle *(* $m_{\mathrm{rest}})$ [Fig. 1(d)], and generate x-ray scattering predictions, $\Delta S_{p_{\mathrm{rest}}m_{\mathrm{rest}}}(q)$ using CRYSOL.^34^ Similarly, we also extract the coordinates from molecular dynamics trajectories of the protein in a modified conformation *(* $p_{\mathrm{exc}})$, within another detergent micelle $\left(m_{\mathrm{exc}}\right)$, and again use CRYSOL to predict $\Delta S_{p_{\mathrm{exc}}m_{\mathrm{exc}}}(q)$ for every value of γ and δ [Fig. 1(e)]. To ensure that the effects of structural fluctuations within the detergent micelle cancel, we next exchanged the coordinates of the detergent micelle to create a set of complementary pdb files that were written with the sampled resting conformations of the protein, $p_{\mathrm{rest}}$, but placed within the sampled micelle conformations of the excited trajectory, $m_{\mathrm{exc}}$, [Fig. 1(f)]; and conversely for the sampled excited conformation of the protein, $p_{\mathrm{exc}}$, placed within the sampled micelle conformations of the resting trajectory, $m_{\mathrm{rest}}$ [Fig. 1(g)]. From these complementary sets of pdb files, $S_{p_{\mathrm{rest}}m_{\mathrm{exc}}}\left(q\right)$ and $S_{p_{\mathrm{exc}}m_{\mathrm{rest}}}\left(q\right)$ were calculated and the expression,

$${\Delta S}_{pm}\left(q\right)=\frac{1}{2}(S_{p_{\mathrm{exc}}m_{\mathrm{exc}}}\left(q\right)-S_{p_{\mathrm{rest}}m_{\mathrm{exc}}}\left(q\right)+S_{p_{\mathrm{exc}}m_{\mathrm{rest}}}\left(q\right)-S_{p_{\mathrm{rest}}m_{\mathrm{rest}}}\left(q\right)),$$ (13)

was utilized to ensure that the term proportional to $\Delta S_{m}(q)$ of Eq. (7) is zero. Figure 3(f) shows the results of these calculations, revealing a continuous evolution of $\Delta S_{pm}(q)$ as γ and δ are varied, and is similar to the smooth variation in x-ray scattering seen for the protein alone [Fig. 3(a)]. In this manner we established a protocol that allows the cross term between the changing protein structure and a detergent micelle to be evaluated from molecular dynamics trajectories, while avoiding the central problem that these predictions become completely dominated by random fluctuations within the detergent micelle. This procedure is therefore utilized for recovering theoretical fits (i.e., best estimates of γ and δ their respective uncertainties) against the experimental data, with further corrections for solvent contrast and micelle fluctuations described below.

### Influence of different sized detergent micelles on difference x-ray scattering

**FIG. 4.**
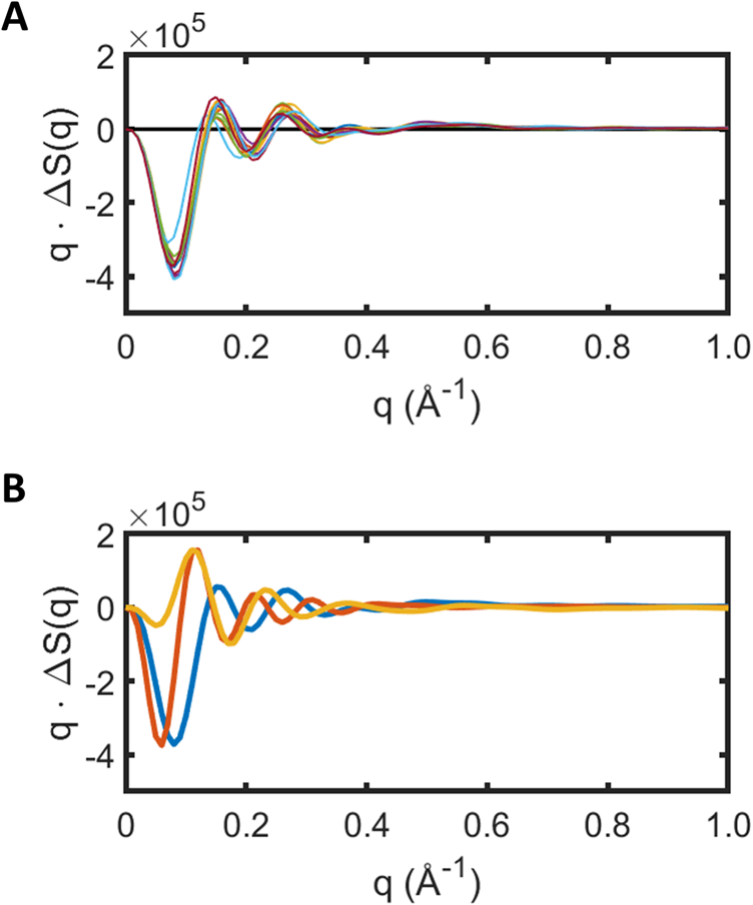
Influence of variations in the number of detergent molecules within a micelle on the predicted difference x-ray scattering curves. (a) Difference x-ray scattering predictions from the protein plus micelle system, $\Delta S_{pm}(q)$, calculated using Eq. (13) with detergent micelles containing 170, 175, 180, 185, 190, 195, 200, 205, 210, 215, 220, 225, and 230 molecules of β-octyl glucoside. (b) The principal SVD component (blue line) and second SVD component (mustard line, scaled by a factor of 100 for comparison) calculated from the data shown in (a). The second SVD component characterizes the changes in difference x-ray scattering due to variations in the size of the micelle. The principal SVD component extracted from detergent micelle fluctuations associated with the final 5 ns of simulations [Fig. 3(e)] is shown for comparison (red line).

Equation (13) provides a working protocol for predicting x-ray scattering changes from a membrane protein within a detergent micelle without these predictions being dominated by random structural fluctuations of detergent molecules. In fitting the difference x-ray scattering data, we used a detergent micelle consisting of 190 molecules of β-octyl glucoside. This number of detergent molecules was chosen since it formed a stable micelle which completely surrounded a single molecule of bacteriorhodopsin without excess molecules breaking off during simulations. This choice, however, is somewhat arbitrary and there will always be a distribution of micelle sizes within any experimental sample. To account for the influence of micelle-size variations, predictions from Eq. (13) were repeated for a selected movement of helices E and F [ γ=1; δ=0, Eq.  (12)] but with the size of the detergent micelle varying from 170 to 230 β-octyl glucoside molecules, increasing in steps of five detergent molecules additional for each trajectory. Figure 4(a) shows that changes in the detergent micelle size cause variation in the predicted $\Delta S_{pm}(q)$, but nevertheless the major features of the difference x-ray scattering are consistent for all micelle sizes. Singular value decomposition using Matlab extracted the mean [principle component, Fig. 4(b), blue line] and variation about the mean [second SVD component, Fig. 4(b), mustard line, scaled 100 fold for visibility] from the x-ray scattering predictions shown in Fig. 4(a). What is striking is that the major variation about the mean in $\Delta S_{pm}(q)$ reflected by the second SVD component is highly correlated [Fig. 4(b), red line; Pearson correlation coefficient for 96% for $q\geq 0.1\;{Å}^{-1}$] with the principal SVD component of micelle-only fluctuations during the last 5 ns of a 20 ns long trajectory [Fig. 3(e)]. Thus, for all practical purposes, if one wishes to apply corrections to the x-ray scattering due to micelle fluctuations [the term of Eq. (7) involving $\Delta S_{m}(q)$, x-ray scattering data represented in Fig. 3(e)] or due to fluctuations associated with changes in the size of the detergent-micelle (extracted as the second SVD component when using Eq. (13) applied to a sequence of trajectories with varying micelle size, Fig. 4), the resulting correction curves are almost indistinguishable for $q\geq 0.1\;{Å}^{-1}$. This observation is presumably due to x-ray scattering changes reflecting the intrinsic length-scale of the detergent micelle in both cases. The inclusion of both terms during minimization would therefore risk a nonphysical situation of both terms having large amplitudes but opposite signs and therefore almost canceling each other. For this reason, we used only one these two corrections during structural fitting [that extracted as the micelle size was varied, mustard line Fig. 4(b)], with the consequence that it is not possible distinguish corrections due to the micelle size fluctuations or those due to micelle structural fluctuations.

### Correction for the solvent excluded volume

**FIG. 5.**
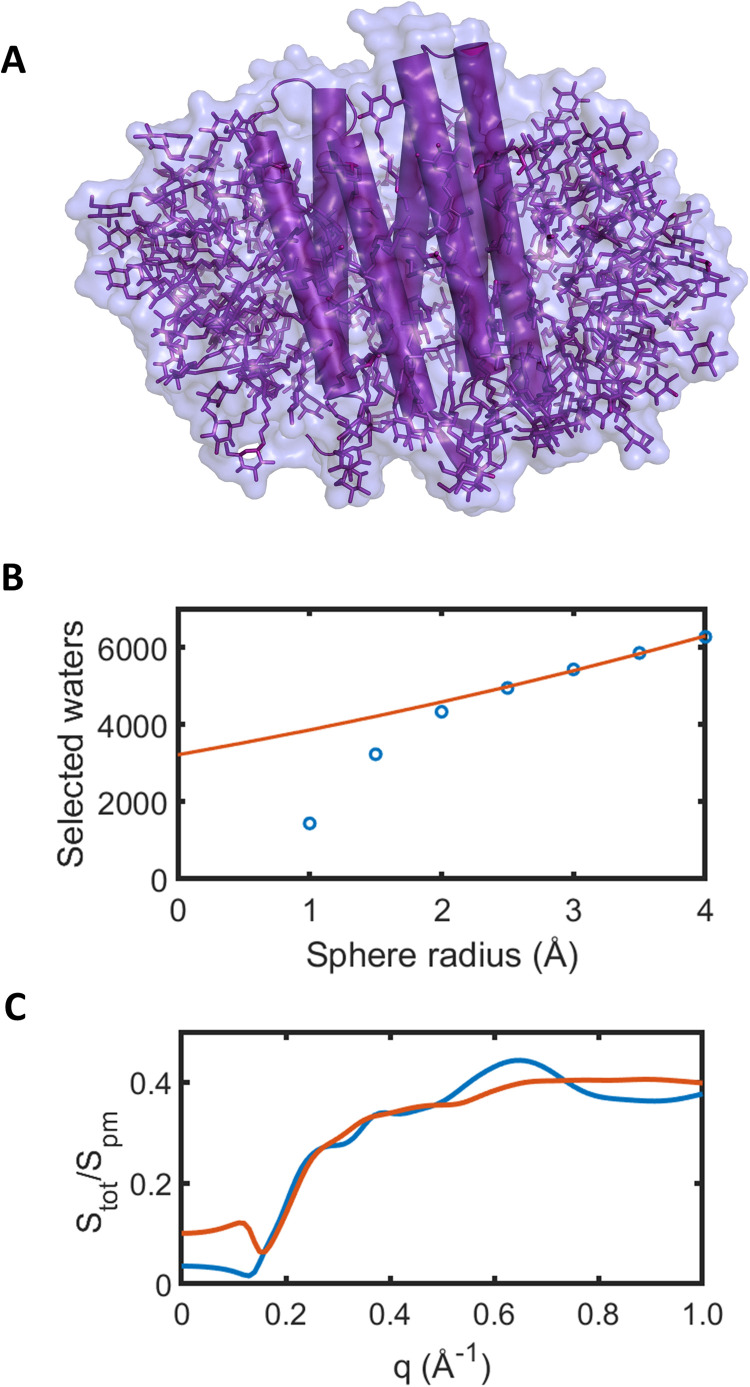
Influence of the solvent excluded volume of the protein and detergent micelle on the predicted difference x-ray scattering curves. (a) Cartoon illustrating how the surface calculated from selected water molecules (transparent surface) used for solvent exclusion calculations fully surrounds the protein and detergent micelle. (c) Comparison of the solvent exclusion correction expressed as the ratio $S_{\mathrm{tot}}(q)/S_{pm}(q)$ [see Eq. (11)] when calculated directly for the protein plus detergent micelle coordinates using CRYSOL (blue line); and from Eqs. (8) and (9) (red line) where CRYSOL is used to calculate $S_{pm}\left(q\right)$ from the protein plus micelle, and $S_{\mathrm{solv}}\left(q\right)$ is calculated from water molecules selected in (a) (blue line is scaled by a factor of 2.1 to best map to the red line). (b) Plot of the number of water molecules selected (blue circles) when using sphere radii from 1.0 to 4.0 Å, whereas 4331 water molecules were selected when using a 2 Å radius, the relative scaling between $S_{\mathrm{solv}}\left(q\right)$ and $S_{pm}\left(q\right)$ in Eqs. (10) and (11) was determined by projecting this plot back to an infinitesimal radius (red line, 0 Å corresponds to 3215 water molecules).

Predictions for light-induced changes in the x-ray scattering from the protein and detergent micelle must be further corrected due to the influence of the solvent excluded volume [Eq. (11)]. CRYSOL predicts the total x-ray scattering after correcting for the solvent excluded volume by incorporating empirical corrections to the x-ray scattering function for individual atoms^34^ and these corrections were developed against a wealth of experimental data from soluble proteins. A detergent micelle typically has a much lower electron density^39^ than proteins^40^ and it was unclear if the empirical solvent excluded volume correction developed for soluble proteins should also be applied to integral membrane proteins within a detergent micelle. We therefore again utilized molecular dynamics simulations in GROMACS to extract the solvent excluded volume correction for the protein plus micelle system. Specifically, in addition to simulations of the protein and micelle within a box filled with water, we ran parallel unrestrained simulations of a box with water alone. We then superimposed the protein plus micelle coordinates upon the simulation with water alone, selected only those water molecules that were within a sphere of 2 Å radius from an atom of the protein or micelle, and wrote out a pdb file of water molecules alone that fully encompassed the protein and detergent [illustrated in Fig. 5(a)]. From Eq. (9), the total scattering after the excluded volume was calculated whereby the term $S_{\mathrm{solv}}(q)$ was extracted from pdb files of only water molecules selected above; and $\text{cos }\Phi_{\mathrm{solv}}$ was calculated according to Eq. (10). When cutting out spheres of water molecules on all atoms in the protein-micelle complex, there will be an over-counting due to some water atoms which protrude beyond the protein's surface. We, therefore, compensated for over counting the selected water molecules by varying the sphere radius between 1 and 4 Å, and counting the number of water molecules at each radii. From this result we created a linear regression to count the number of water molecules at 0 Å [Fig. 5(b)] and the amplitude of $S_{\mathrm{solv}}(q)$ was adjusted accordingly. Although the amplitude of the applied correction for $S_{\mathrm{tot}}(q)/S_{pm}(q)$ was sensitive to the details of this procedure, both the GROMACS and the CRYSOL protocols yielded very similar shaped corrections curves for $q\geq {0.15\;Å}^{-1}$ [Fig. 5(c)] and consequently structural fitting (which treats the overall scaling as a parameter to be optimized) is not very sensitive to those details. Below $q={0.15\;Å}^{-1}$, the GROMACS approach predicts a smaller contrast between the protein and micelle and, as such, the approach developed here provides a low-angle correction when considering a protein-micelle complex.

### Convolution with the undulator spectrum

Since polychromatic radiation was used to probe light-induced structural changes in bR, it was essential to account for the effect of the undulator spectrum on the x-ray scattering predictions. This effect was incorporated into the analysis by interpolating all x-ray scattering predictions onto a variable *q*-axis which varied inversely with the x-ray energy, and summing these interpolated x-ray scattering predictions according to the amplitude of the undulator spectrum for any given x-ray energy.

### Structural fitting to the experimental data

Having developed the above tools and approximations, we searched for a best fit to the first and second experimental basis spectra [Fig. 2(b)] by varying the Cα perturbations according to the parameters γ and δ of Eq. (12). As in our earlier approaches,^12,18,24,25^ structural refinement minimized the function,

$$R=\frac{\sum \sqrt{{\left({w\left(q\right)\cdot (A_{1}\cdot \Delta S}_{\mathrm{theory}}\left(q\right)-{\Delta S}_{\mathrm{expt}}\left(q)\right)\right)}^{2}}}{\sum \sqrt{{\left({w\left(q\right)\cdot \Delta S}_{\mathrm{expt}}\left(q\right)\right)}^{2}}},$$ (14)

for each of the two basis spectra [states 1 and 2, Fig. 1(b)]; w(q) is a weighting factor introduced to emphasize regions where the structural content of the difference x-ray scattering data are strongest; and ${\Delta S}_{\mathrm{expt}}\left(q\right)$ is the experimental data for each of the two states. The term ${\Delta S}_{\mathrm{theory}}\left(q\right)$ is extracted for each value of γ and δ [Eq. (10)] using the following expression:

$$\begin{array}{cc} {\Delta S}_{\mathrm{theory}}\left(q\right)=\frac{S_{\mathrm{tot}}(q)}{S_{pm}(q)}\cdot \text{exp}(-\frac{B_{p}q^{2}}{16\pi^{2}})\cdot [\Delta S_{p}(q)+\frac{1-\text{erf}(q_{pm},\Delta q_{pm})}{2} & +\frac{1-\mathrm{erf}(q_{pm},{\Delta q}_{pm})}{2}\times \{\Delta S_{pm}(q)-\Delta S_{p}\left(q\right)+A_{2}\cdot \;\Delta S_{mc}(q)\}] \end{array},$$ (15)

where the ratio $S_{\mathrm{tot}}(q)/S_{pm}(q)$ is used to correct for the influence of the solvent excluded volume [Fig. 5(c), Eq. (11)]; where $\Delta S_{p}(q)$ is calculated directly from the atomic coordinates for the protein extracted from restrained molecular dynamics trajectories 5 ns in duration [Fig. 3(a)]; $\Delta S_{pm}(q)$ is extracted from the atomic coordinates of the protein and micelle using Eq. (13) [Fig. 3(f)]; $B_{p}$ is introduced in analogy to the crystallographic B-factor^12^ and reflects a loss of scattering power at higher angle arising from fluctuations about the mean atomic positions of the atoms of the protein; erf is the error function and the parameter $q_{pm}$ was varied during structural refinement and ${\Delta q}_{pm}$ was set as 25% of $q_{pm}$, and this parameter quantifies the x-ray scattering angle above which the change in x-ray scattering from the micelle may become negligible due to the highly disordered nature of the detergent micelle; and Δ $S_{mc}(q)$ is a micelle correction term introduced to account for either low-angle corrections due to micelle fluctuations [Fig. 3(e)] or first order-corrections to the protein-micelle scattering term due to variations in the number of detergent molecules per micelle [Fig. 4(b), used here throughout structural fitting] and the amplitude, $A_{2}$, was also varied during refinement. For any given 5 ns restrained molecular dynamics trajectory, 100 structural pairs (of 501) were selected with the highest Pearson-correlation scores calculated relative to the experimental data over the domain $q\geq 0.20\;{Å}^{-1}$, and Cα movements associated with these 100 pairs were used in quantifying the most probable conformational changes. Overall, six parameters were varied during refinement ( $\gamma ,\;\delta ,\;A_{1},\;A_{2},\;B_{P},\;q_{pm})$, with $A_{1}$ optimized for both states simultaneously and, as noted below, it was not necessary to optimize $B_{p}$.

**FIG. 6.**
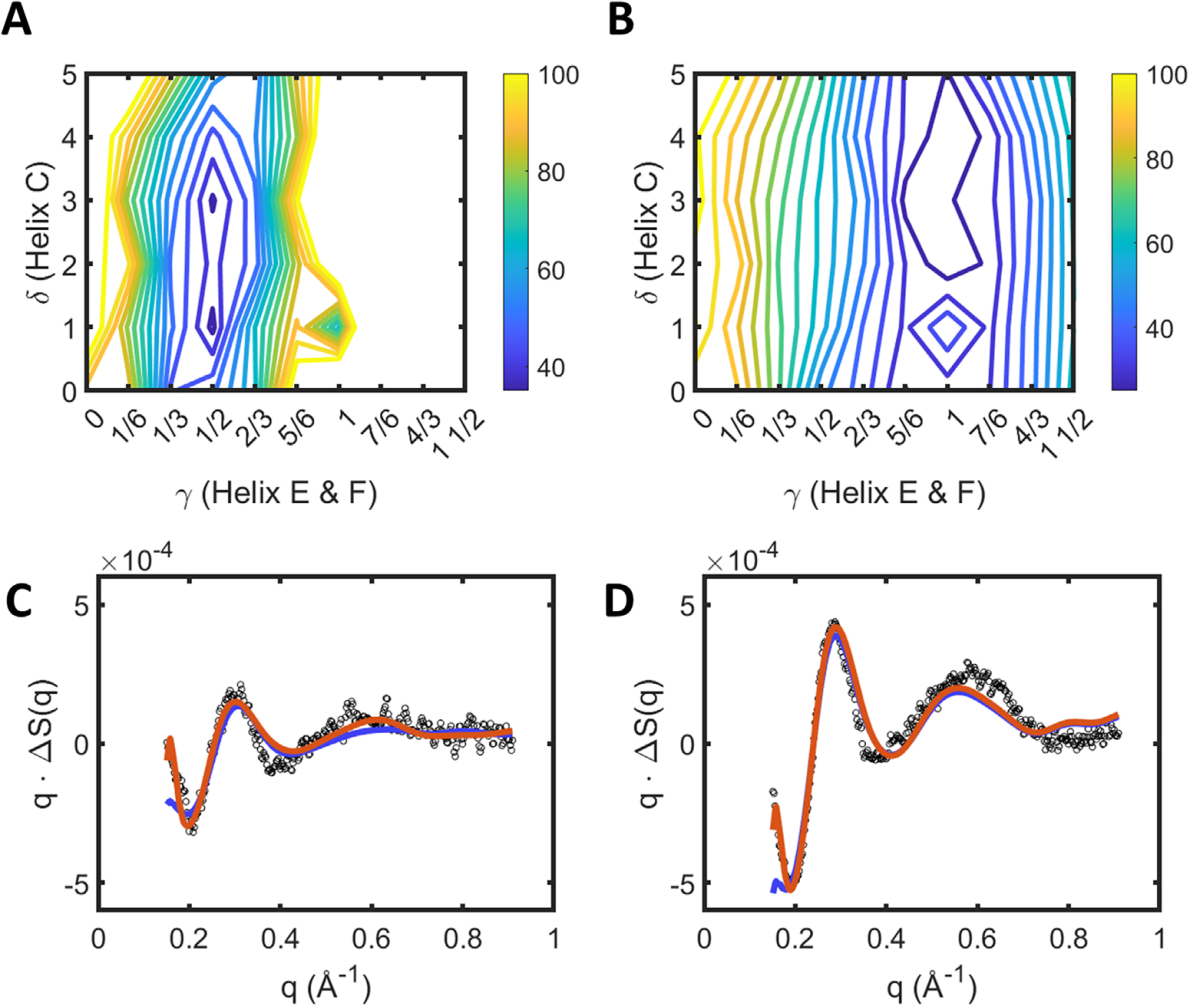
Results of structural fitting against difference x-ray scattering data when minimizing the R-factor [Eq. (14)] using the model summarized in Eq. (15). Protein conformations were sampled from restrained molecular dynamics simulations of 5 ns in duration, where Eq. (12) defines γ and δ. (a) Contour surface showing the R-factor recovered for a given displacement of helices EF ( γ) and helix C ( δ) [Eq. (12)] when fitted against the state 1 basis spectrum [Fig. 2(b), blue line]. (b) Contour surface showing the R-factor recovered for a given displacement of helices EF ( γ) and helix C ( δ) when fitted against the state 2 basis spectrum [Fig. 1(b), black line]. All fits in panels (a) and (b) apply the same (optimal) scaling ratio, $A_{1}$, defined in Eq. (14). (c) Best-fit x-ray scattering prediction (red line) superimposed upon the experimental data (black circles) for state 1. The blue line resulted when the fitting procedure was repeated without micelle and cross term corrections. (d) Best-fit x-ray scattering prediction (red line) superimposed upon the experimental data (black circles) for state 2. Experimental data were originally presented in Ref. 12. The blue line resulted when the fitting procedure was repeated without micelle and cross term corrections.

**FIG. 7.**
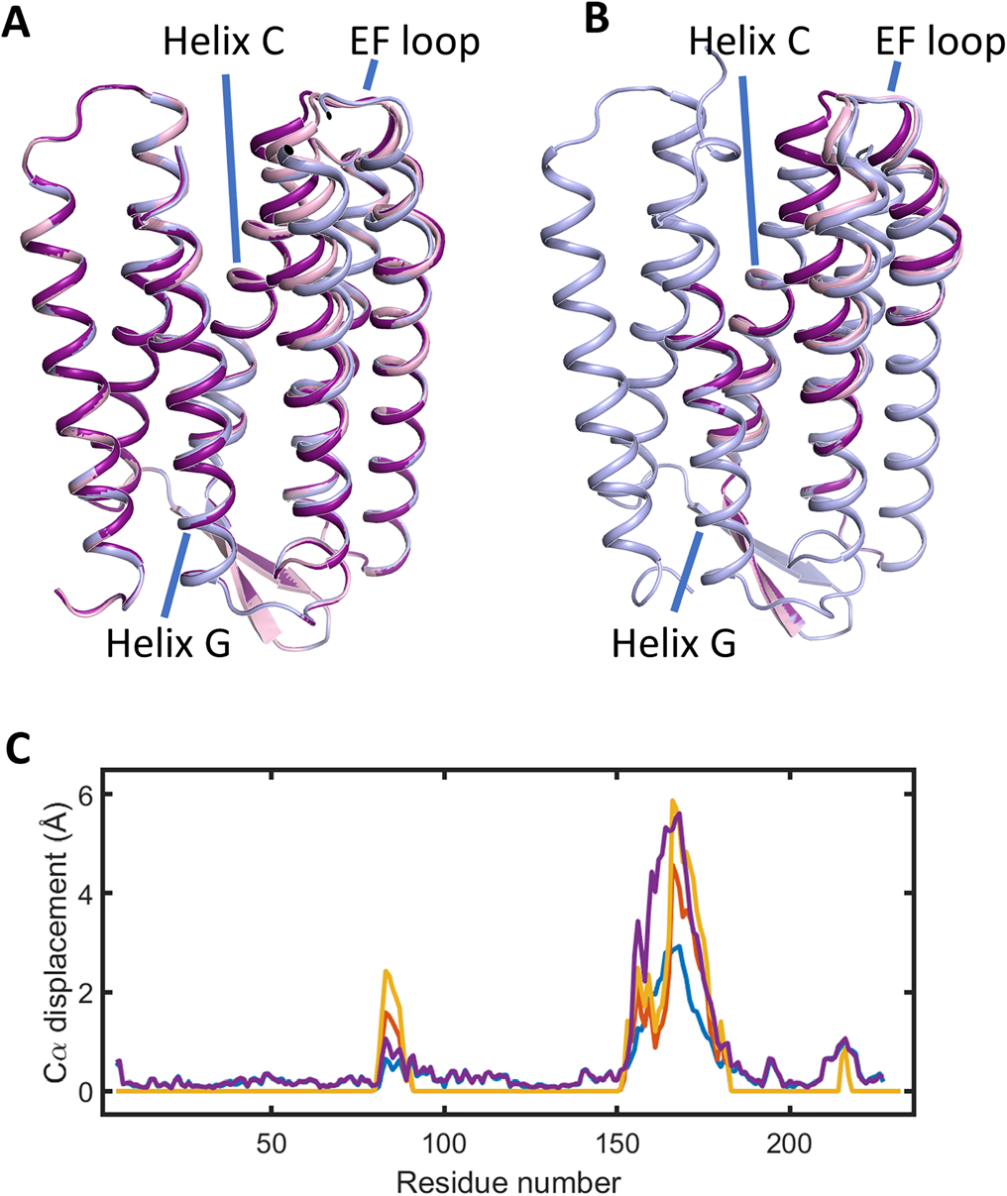
Light-induced conformational changes in bR predicted by fitting against the difference x-ray scattering basis spectra. (a) Structural results from the fitting protocol developed here. The bR resting conformation is illustrated in purple; the best-fit model for state 1 is illustrated in pink; and the best-fit model for state 2 is illustrated in gray/blue. (b) Structural results from the fitting protocol previously developed in Ref. 12. The bR resting conformation is illustrated in purple; the best-fit model for the intermediate conformational state in Ref. 12, is illustrated in pink; and the best-fit model for the late conformational state in Ref. 12 is illustrated in gray/blue. (c) Light-induced displacements of C in Cα-atoms for the two modeling protocols. The amplitude of the atomic displacement of Cα-atoms is plotted vs residue number for state 1 (blue) and state 2 (purple) using the fitting model developed here; and the intermediate (mustard) and late (red) conformational states of Ref. 12.

Results of fitting to the experimental data using this procedure are illustrated in Figs. 6(a) and 6(b) as a contour plot showing the changes in R-factor [Eq. (12), calculated with the weighting function $w\left(q\right)$ set to unity for all q] as γ and δ are varied for states 1 and 2, respectively. The minima in these contour plots are identified for state 1 at γ=0.5 ±  0.2 and δ=3 ± 2.5, where the uncertainty is estimated as the change needed to increase the R-factor by 25% of R-minimum on the contour plot [Figs. 6(c) and 6(d)]. For state 2, the corresponding values were γ= 1.0 ± 0.2 and δ=3 ± 1.5. Whereas the parameter δ describing the movement of helix C seems large and very poorly defined, from the selected sub-set of optimal structures we find that the mean Cα displacements on helix C were only 0.4 ± 0.3 Å and 0.7 ± 0.3 Å for state 1 and state 2, respectively, when calculated from residues 81 to 89 of helix C. By comparison, the crystallographic structures 5B6Z vs 5B6V gives a mean Cα displacement of 0.46 Å for these residues of helix C. Similarly, the mean Cα displacement from residue 152 of helix E to residue 182 of helix F were 1.6 ± 0.6 Å and 3.0 ± 0.6 Å (Fig. 7) when calculated for the sub-set of optimal structures for state 1 and state 2, respectively. For comparison, pdb entry 6RPH vs 6RQP gives a mean Cα displacement of 3.4 Å (after adjustments for disordered regions described above) for the selected residues of helices E and F.

The micelle damping parameter $q_{pm}$ was refined as 0.129 Å^−1^ as this value led to the lowest R-factor [Eq. (14)], and this value which can be equated with a B-factor of 8000 Å^2^. Using $B=8\pi^{2}\langle u^{2}\rangle$, where u is the amplitude of mean atomic displacement about the median position, this estimates random atomic motions within the membrane as the order of 10 Å, which is compatible with the motions of detergent micelles during the course of a simulated trajectory. By contrast the best fit was recovered when $B_{p}$, the damping parameter for the protein alone, was made arbitrary small and it was not optimized further. We, therefore, conclude that the structural fluctuations within the protein were quite well described by structural variations sampled during the Cα-restrained molecular dynamics simulations and the fitting routine did not benefit from the parameter $B_{p}$, which was otherwise essential when fitting using rigid-motions.^12^

Figures 6(c) and 6(d) superimpose the best-fits from this procedure onto the experimental basis-spectra. Optimal predictions for ${\Delta S}_{\mathrm{theory}}\left(q\right)$ from Eq. (15) [red line, Figs. 6(c) and 6(d)] appear to capture all aspects of the experimental data ${\Delta S}_{\mathrm{expt}}\left(q\right)$ for both basis spectra for state 1 and 2. However, the signal-to-noise ratio for state 2 is better than that of state 1, and this reflects in the R-factor [Eq. (15)] of 33.5% and 20.4% being recovered for states 1 and 2, respectively, when using a weighting of unity over the q-domain 0.15 Å≤q≤0.9 Å over which the structural fitting was performed. If we remove the micelle-correction terms deriving from Eq. (13) [Fig. 3(f)] and that emerging from variations in the detergent micelle structure [Fig. 4(b)], then the resulting fits at lower x-ray scattering angle are compromised [red line, Figs. 6(c) and 6(d)] with the R-factors increasing to 52.5% for state 1 and 29.6% for state 2. For comparison, the R-factors reported in Ref. 12 for the intermediate and late states were 32% and 28% respectively, but these were calculated over the smaller q-domain 0.19 Å≤q≤0.7 Å.

## DISCUSSION

Over the last-decade, TR-XSS studies of membrane protein structural changes have become a mature experimental method.^12,18,24,25,28,29^ Despite variations in detergent concentrations, variations in photo-excitation levels, difference in signal-to-noise ratios, and other factors associated with sample preparation or the execution of an experiment, the measured difference XSS basis spectra from light-sensitive integral membrane proteins are highly reproducible [Fig. 2(b)]. A sequence of time-dependent difference-XSS measurements from detergent solubilized membrane proteins [Figs. 2(c) and 2(d)] reveal if secondary structural changes occur and it is usually quite straightforward to extract the rise and decay times for a sequence of structural states. While structural models have been presented from this body of work, the theoretical foundations underpinning the modeling of different XSS data from integral membrane proteins have been somewhat glossed over due to challenges associated with detergent micelles that both fluctuate dramatically and often scatter x-rays more strongly than the protein of interest.

This analysis explicitly addresses this challenge by creating a framework that allows an atomistic description of the protein and detergent micelle using the molecular dynamics package GROMACS.^38,41^ As previously, the first step is to identify candidate motions of secondary structural elements. In this analysis, these motions were extracted from recent time-resolved serial crystallography studies of bacteriorhodopsin,^31,32^ which makes bacteriorhodopsin a particularly favorable case. It is, however, possible to identify candidate motions from molecular dynamics trajectories^13–15,21,23,24,27–29^ or potentially building approximate models using experimental results from other biophysical methods.^42^ Using selected candidate motions to guide molecular dynamics simulations, we identified how structural fluctuations within the detergent micelle influenced the different x-ray scattering curves [Figs. 3(b)–3(e)] and how changes in the detergent micelle size influenced the difference x-ray scattering (Fig. 4). Since both corrections were similar, they were incorporated as a single micelle correction to the predicted x-ray scattering [Δ $S_{mc}(q)$ of Eq. (15)]. The most important conceptual advance of the new approach was to interchange the atomic coordinates of the protein micelle when comparing molecular dynamics trajectories for resting and photo-activated structures [Figs. 1(d)–1(g)]. This allowed a formalism to be established in which the protein-micelle x-ray scattering cross term could be determined without being dominated by structural fluctuations within the micelle [Eq. (13), Fig. 3(f)]. Damping corrections were introduced for the protein-micelle systems [Eq. (15)], solvent contrast corrections were required (Fig. 5), and the x-ray spectrum of the pink-beam undulator had to be explicitly incorporated into the analysis, all of which are consistent with our earlier formalism.^12,18,24,25^

Structural conclusions drawn from this approach to molecular fitting of difference x-ray scattering data (Fig. 6) are largely consistent with those previously reported (Fig. 7). The mean Cα displacement helix C was found to be 0.4 ± 0.3 Å and 0.7 ± 0.3 Å for state 1 and state 2 in this structural analysis, whereas our previous fitting against TR-XSS data^12^ yielded the values 0.9 and 1.3 Å for the intermediate and late conformational states, respectively. It seems plausible that the uncertainties in the amplitude of this displacement are large since the Cα coordinate fluctuations throughout the protein within our restrained molecular dynamics simulations are the order of 0.2 Å (Fig. 6). For the movement of the cytoplasmic portions of helix E and F, we recovered a mean Cα displacement of 1.6 ± 0.6 Å and 3.0 ± 0.6 Å for state 1 and state 2, respectively, whereas we previously concluded that these amplitudes were 2.0 and 2.6 Å for the intermediate and late conformational states of bR.^12^ We suggest that agreement within uncertainties in the displacement associated with state 2 and the earlier late conformational state is acceptable.

Time-resolved serial crystallography^4,5^ is a rapidly growing field of research and TR-XSS has been established for more than a decade.^11^ The theoretical approach presented here underpins earlier assumptions when working with integral membrane proteins^12,18,24,25,28,29^ that the presence of detergent micelles does not necessarily affect structural fitting protocols that focus attention upon data in domain $q\geq 0.2{\;Å}^{-1}$. Moreover, this analysis improves the fit to low scattering angle data [Figs. 5(e) and 5(f)] and justifies the assumption that this region is dominated by the influence of the detergent micelle. With the widespread use of rapid x-ray detectors at synchrotron radiation sources^43^ and the development of appropriate microfluidic mixing technologies for reaction initiation, it should become possible to extend the field of different XSS studies of integral membrane proteins to incorporate protein and substrate or reagent mixing studies to initiate time-resolved studies. Since caged compounds have also been successfully used for such studies,^28,29^ the sub-field of TR-XSS has the potential to grow beyond the study of naturally light-driven systems. Because all future experimental developments require solid theoretical foundations and because the detergent micelle may itself be influenced by mixing, the analysis tools and fitting protocols developed here should provide a framework that allows the field to look to the future with confidence.

## MATERIALS AND METHODS

### Sample preparation

#### Sample preparation

bR was solubilized in β-octylglucoside and concentrated to 1.3 mM, as judged from the bR absorption peak at 570 nm, as described in more detail in earlier studies.^12^

#### TR-XSS data acquisition and processing

TR-XSS data were collected at beamline ID09 of the European Synchrotron Radiation Facility (ESRF). Samples bR concentrated to 35 mg/ml were delivered across a polychromatic x-ray beam produced from an x-ray undulator (18 keV, pink beam, ΔE/E ≈ 4%) through a quartz capillary (0.5 mm diameter, Hampton Research). Samples were driven using a motorized syringe pump (neMESYS) with a flow rate of 3 *μ*l/s. X-ray pulse trains of 1 or 5 *μ*s in duration were isolated from a continuous filling of the storage ring using an x-ray chopper. X-ray scattering images were recorded on a Rayonix MX170-HS detector (1920 × 1920 pixels, pixels binned 2 × 2 to a pixel-size of 88.5 *μ*m) located 350 mm from the sample position. X-ray scattering data were collected at room temperature (∼20 °C). Data were merged over the domain 0.025 Å^−1^ < *q *<* *3.0 Å^−1^.

Samples of bacteriorhodopsin were photo-activated using a 532 nm laser pulse 4 ns in duration with 500 *μ*J/pulse focused into a spot size of 0.31 × 1.7 mm^2^ (full width half maximum). The triggering and timing of the green laser pulse and the x-ray detector were integrated into the beamline control system. X-ray scattering data were integrated in concentric rings^12^ prior to normalization about the isosbestic points at *q *=* *1.6 Å^−1^. Outlier rejection was performed in two steps: the ring-integrated absolute scattering curves were rejected if they deviated more than 10% from the median value in the q-range 2.0–2.5 Å^−1^, followed by an outlier rejection scheme where difference scattering curves (ON minus OFF) where rejected if they deviated by more than three standard deviations from the mean of a given run. Typically, 4%–10% of all the data were rejected, depending upon the experimental run. The influence of laser-induced heating was measured and removed using data recorded using an IR laser (λ = 1470 nm) to heat the sample. At this wavelength heat was deposited, the IR light did not photo-isomerize the retinal chromophore. Heating data were collected and were processed in the same fashion as described for the photo-activated datasets. Heat free light-induced difference scattering data were then computed by scaling and removing the thermal signal from each time-point, where the scaling factor is chosen to minimize the difference x-ray scattering differences over the domain $0.51\;{Å}^{-1}\leq q\leq 1.45\;{Å}^{-1}$. Data after heat-removal are presented in Figs. 2(c) and 2(d)and were analyzed using two-component fitting based upon an exponential decay of the first component and a complementary rise of the second component. Experimental details for the 2009 studies performed at the same beamline have been described previously.^12^

#### Molecular dynamics simulations using GROMACS

Monomeric bacteriorhodopsin protein/micelle complexes were built into a β-octylglucoside membrane using CHARMM-GUI Micelle Builder.^44,45^ A range of protein/micelle complexes were built, including 170, 175, 180, 185, 190, 195, 200, 205, 210, 215, 220, 225, and 230 β-octylglucoside molecules, with simulations using 190 β-octylglucoside molecules being preferred for most of the analysis. Lipid ring and protein surface penetration was checked by CHARMM as part of the system building options. The system was built within a solvent box consisting of 20 Å in all dimensions, using three-site CHARMM modified TIP3 water model and a concentration of 0.15 M of NaCl. GROMACS input files were created by CHARMM.^46,47^ GROMACS 2019.1^37,38^ was used to run restrained molecular dynamics simulations of bR using the CHARMM36 force-field.^48^ Energy minimization was carried out first using LINCS constraints algorithm with constraints on hydrogen bonds. The second stage was equilibration of the system in six steps with energy and dihedral restraints on the β-octylglucoside molecules. No movement of the protein backbone was allowed during the equilibration and production steps. Molecular dynamics trajectories of 20 ns in duration (preparative runs) or 5 ns (fitting runs) were used to produced 2000 or 500 pdb files, respectively, each pdb file separated by 10 ps. These pdb files were used to calculate the theoretical scattering terms for protein, membrane, and protein plus membrane. Un-restrained molecular dynamics simulations involving only water were also performed using standard settings in GROMACS 2019.1. Theoretical difference x-ray scattering curves were calculated using CRYSOL.^34^ Data and in-house code are available at Ref. 49.

## Data Availability

The data and in-house code that support the findings of this study are openly available in GitHub at https://github.com/Neutze-lab/TR_XSS, Ref. 49.
